# Polymorphic post-transplant lymphoproliferative disorder in a gilt

**DOI:** 10.1080/01652176.2019.1661542

**Published:** 2019-10-01

**Authors:** Giorgia Tura, Valeria Pellegrino, Giancarlo Avallone, Francesca Barone, Maria Laura Bacci, Riccardo Villa, Alessandro Spadari, Domenico Ventrella, Francesco Dondi, Valeria Corradetti, Gaetano La Manna, Giuseppe Sarli

**Affiliations:** a Department of Veterinary Medical Science, University of Bologna, Ozzano Emilia, BO, Italy;; b Cellular Substrates, The Lombardy and Emilia Romagna experimental Zootechnic Institute (IZSLER), Brescia, Italy;; c U.O. Nephrology, Dialysis and Transplantation Unit, Sant’Orsola-Malpighi University Hospital, Bologna, Italy

**Keywords:** Swine, post-transplant lymphoproliferative disorder, polymorphic, porcine endogenous retroviruses

In a study aimed at identifying early biomarkers of renal rejection, a 4-month-old commercial gilt weighing 60 kg underwent unilateral nephrectomy and a subsequent renal transplantation. The study was approved by ministerial authorization number 279/2013- B.

The gilt belonged to a caseload of 18 pigs of the same age and genetic, that underwent unilateral nephrectomy to receive a renal graft from a randomly selected donor of the same age and same blood group.

After having been explanted, the kidney from donor was maintained on ice and perfused with sterile and cold Ringer’s lactate solution, following insertion of separate cannulas into the renal vein and artery until it was implanted in the receiving animal. Then, the kidney was positioned in inguinal position and renal artery and vein of the grafted kidney were anastomosed respectively with iliac artery and vein of the receiving pig. Two surgical teams working simultaneously allowed blood reperfusion of the grafted kidney in less than 1 h.

Pharmacological immunosuppression was achieved by methylprednisolone (Urbason, Sanofi, Milan, Italy) administered intravenously at a dosage of 1 mg/kg BW/daily for the first 7 days combined with tacrolimus (Prograf, Astellas Pharma, Milan, Italy) for the whole experimental period (4 weeks). Tacrolimus was administered intravenously at an incremental dose starting from 0.05 to 0.3 mg/kg BW/daily to ensure a blood tacrolimus concentration of 45–50 ng/mL. During the experiment, several consecutive blood samples were collected, including the one before euthanasia, which is reported in Table 1, and all showed values within normal ranges. Thirty days after transplantation, the animal was euthanized and underwent necropsy.

**Table 1.** Results of the blood analysis of a sample collected before suppression.

Variable	Unit	Result	Reference interval
Hemoglobin	gr%	6.8	10–14
Hematocrit value	%	21.5	35–45
RBCs	cells/mm³	5,380,000	4,900,000–7,600,000
MCV	fL	39.9	57–70
MCHC	gr%	31.5	29–35
RDW	%	23.7	14–17
Platelets	cells/mm³	220,000	200,000–500,000
MPV	fL	6.4	6–14
WBCs	cells/mm³	7180	9800–21,000
Lymphocytes	%	59.8	34–60
Monocytes	%	8.9	2.5–6.5
Neutrophils	%	28.1	25–55
Eosinophils	%	2.0	1.5–15
Basophils	%	0.5	0.5–1.5
Lymphocytes	cells/mm³	4300	4500–12,000
Monocytes	cells/mm³	640	350–1500
Neutrophils	cells/mm³	2020	3000–12,000
Eosinophils	cells/mm³	140	200–2800
Basophils	cells/mm³	30	0–1500

The values are within normal ranges as were those sampled during the experiment.

RBCs: Red blood cells; WBCs: White blood cells; MCV: mean corpuscular volume; MCHC: mean corpuscular hemoglobin concentration; RDW: Red blood cells distribution width; MPV: mean platelet volume.

After necropsy tissue samples were collected, fixed in 10% buffered formalin and paraffin embedded. Subsequently, tissues were routinely processed for histologic evaluation. Immunohistochemistry was performed on formalin fixed paraffin embedded sections. Three-micrometer-thick sections were dewaxed and rehydrated. Endogenous peroxidase was blocked by immersion in H_2_O_2_ 0.3% in methanol for 30 min. For antigen retrieval, sections were immersed in 200 mL citrate buffer (pH 6.0) and heated in a microwave oven at 750 W for two 5-min cycles for CD3 immunostaining while in 200 mL EDTA buffer (pH 8.0) and heated in a microwave oven at 750 W for two 5-min cycles for CD79a immunostaining. After microwave treatment sections were allowed to cool at room temperature for approximately 20 min. Slides were incubated with the primary antibody overnight at 4 °C (CD3: mouse, monoclonal, clone F7.2.38; dilution 1:60; Dako, Glostrup, Denmark; CD79: mouse, monoclonal, clone HM57 dilution 1:750; Santa Cruz Biotechnology, Segrate [MI], Italy). The reaction was revealed by a commercial streptavidine-biotine-peroxidase technique (ABC kit elite, Vector, Burlingame, CA, USA) and visualized with 3-amino-9-ethylcarbazole (Dako, Glostrup, Denmark). Slides were counterstained with Mayer’s hematoxylin. A positive control section of swine hyperplastic lymph node was used for both antibodies. As a negative control, the primary antibody was replaced with an irrelevant, isotype-matched antibody to control for nonspecific binding of the secondary antibody.

Real time polymerase chain reaction (qPCR) and gel-based PCR were performed after necropsy only in the post-transplant lymphoproliferative disorder (PTLD)-affected gilt. The first was used to detect the presence of PERVs and the second to detect PLHV-1 according to previously published methods (Argaw et al. 2002 and Santoni et al. 2006, respectively).

Several lymph nodes like tracheobronchial (Figure 1(A)), lombo-aortic (Figure 1(B)), renal, splenic (Figure 1(C)), gastric and mesenteric (Figure 1(F)) appeared from mild to markedly enlarged (up to 5 cm in length, mainly the splenic, gastric and mesenteric), edematous, pale and the cut surfaces were white, homogeneous and firm. In the thorax, aortic, tracheobronchial and mediastinic lymph nodes were severely enlarged.

**Figure 1.**
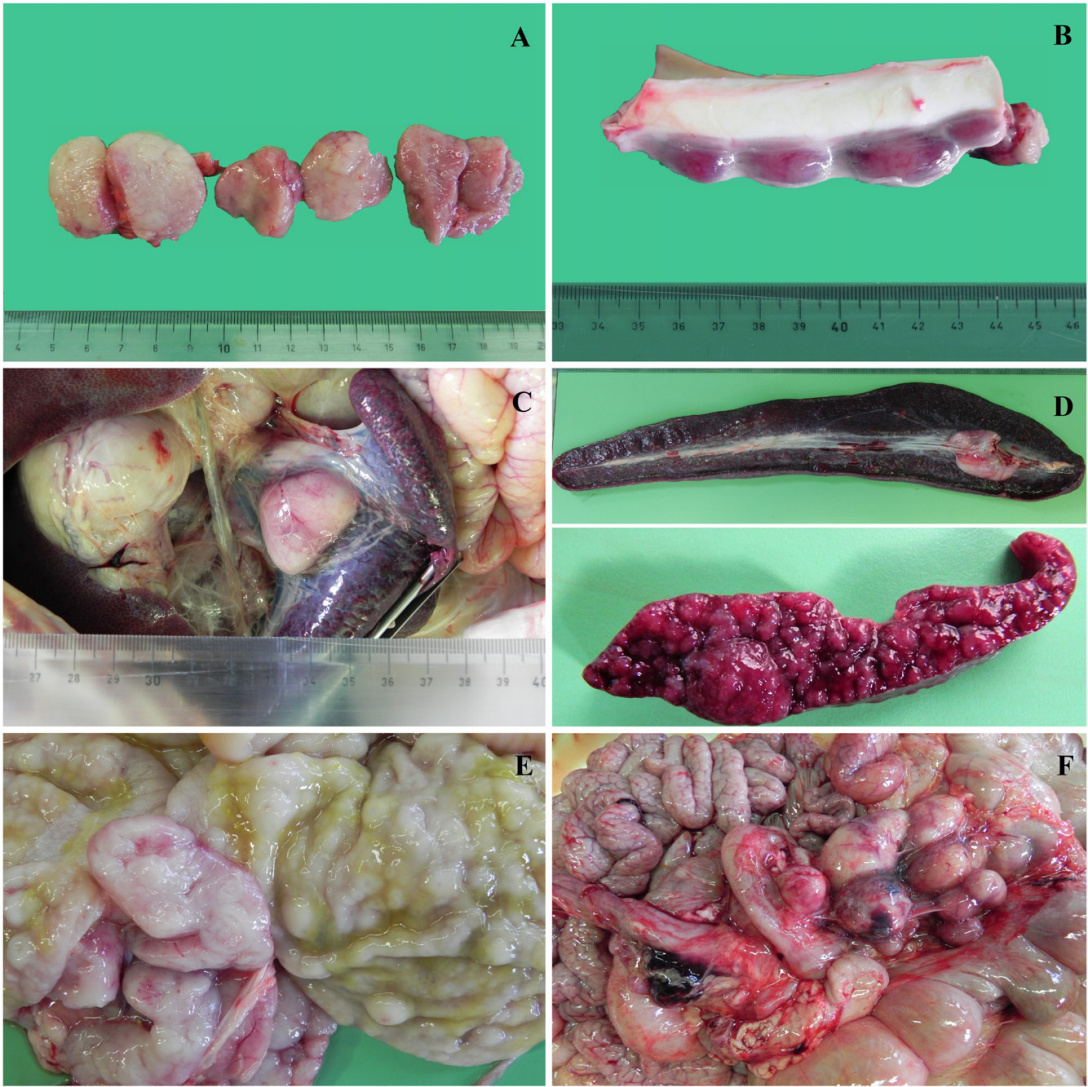
Severe lymphadenomegaly of the tracheobronchial (A), lombo-aortic (B) and splenic (C) lymph nodes. Moderate enlargement of the spleen and severe diffuse multiple bulging nodules (D). Gastric mucosa with diffuse nodular proliferation up to 2 cm in diameter (E). Severe enlargement of the mesenteric (F) lymph nodes.

Microscopically, in the lymph nodes, the architecture was completely effaced by neoplastic tissue (Figure 2(A,B)). The neoplasm was composed of densely cellular sheets of round cells, supported by scant fibrovascular stroma. Neoplastic cells were large (more than 3 erythrocytes), 40–50 μm in diameter, with distinct cell borders, high nuclear to cytoplasmic ratio and scant eosinophilic cytoplasm. Nuclei were round and paracentral with finely stippled chromatin and 2–3 basophilic nucleoli. Pleomorphism was prominent. Mitosis was 10 per high power field. Intermixed between neoplastic cells there were numerous small lymphocytes and plasma cells.

**Figure 2.**
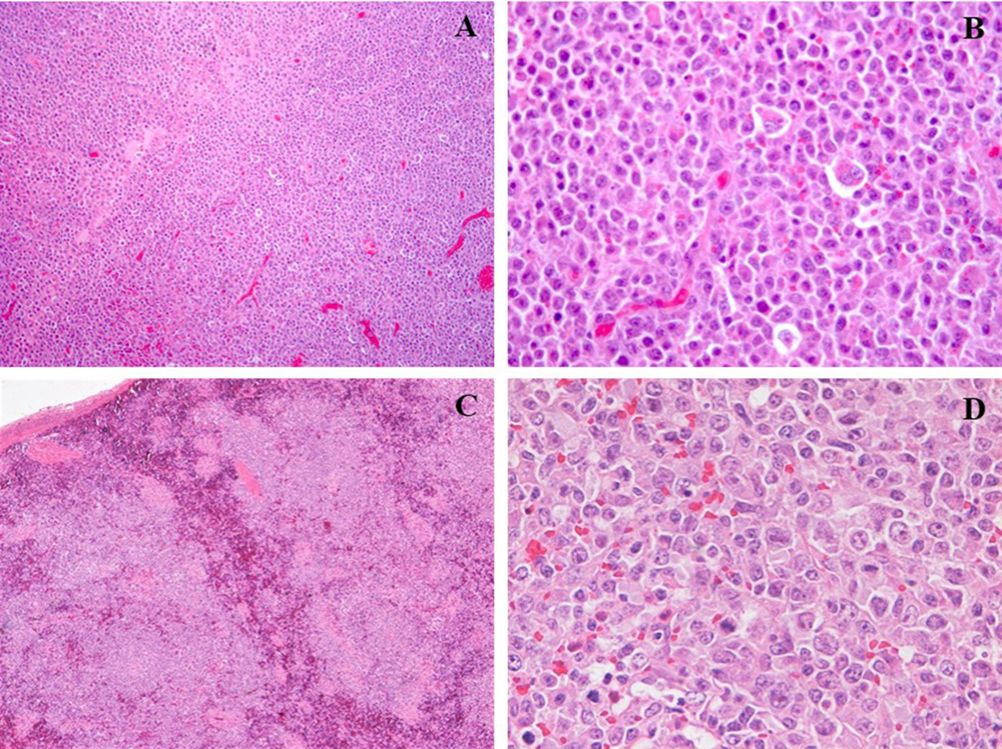
Renal lymph node: sheets of large neoplastic lymphoid cells supported by fine fibrovascular stroma (A, H&E, 10x). Neoplastic large cells with distinct cell borders, high nuclear to cytoplasmic ratio, scant eosinophilic cytoplasm, round and paracentral nuclei with finely stippled chromatin and 2–3 basophilic nucleoli. Intermixed are small round cells with high nuclear to cytoplasmic ratio consistent with small lymphocytes (B, H&E, 40x). Spleen: multifocal to coalescing aggregates of neoplastic lymphocytes intermingled with remnants of red pulp (C, H&E, 10x). Sheet of neoplastic lymphoid cells (D, H&E, 40x).

The spleen was moderately enlarged and on cut surface white pulp appeared as multiple round prominent and bulging nodules, 0.5 to 1.2 cm in diameter (Figure 1(D)). Splenic architecture was replaced by nodular and coalescing aggregates of neoplastic lymphocytes intermingled with remnants of red pulp (Figure 2(C,D)).

Gel-based PCR failed the identification of PLHV-1 genome in lymph node samples while the presence of PERVs was assessed by qPCR. Melting peaks for the lymph node samples confirmed the presence of the retroviruses at 80.5 ± 0.5 °C (Figure 3). 

**Figure 3.**
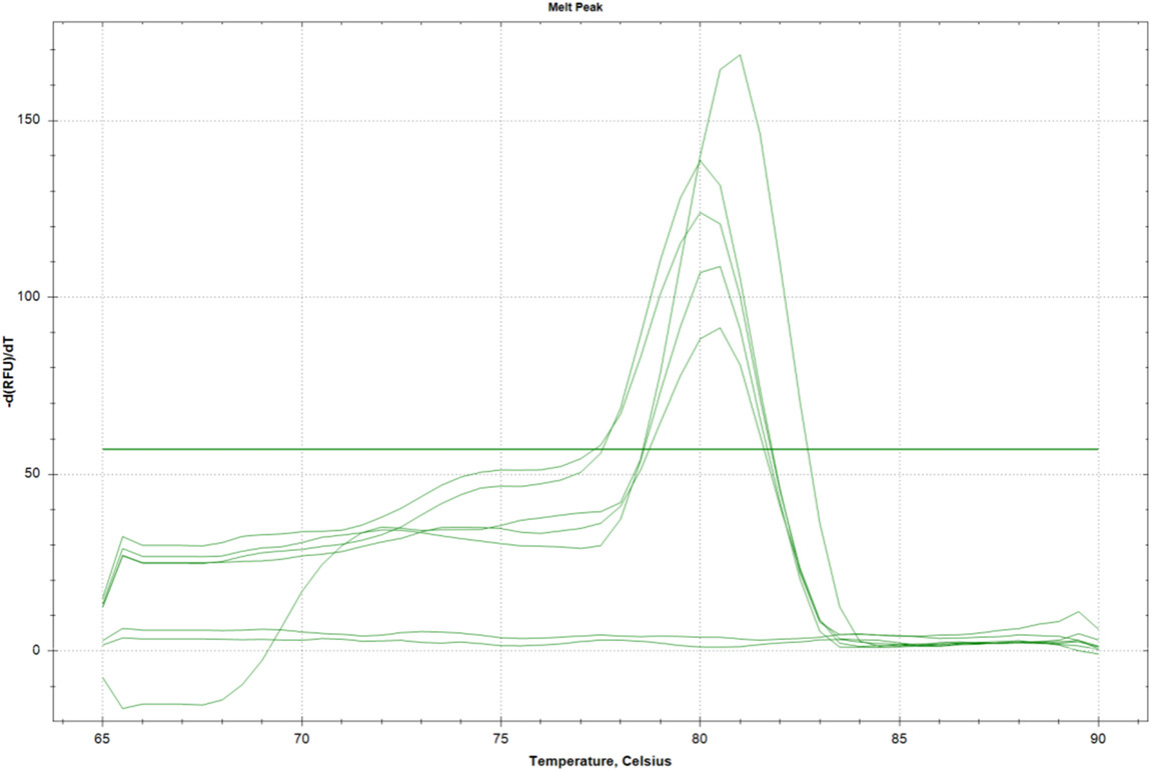
qPCR results. Melting peaks (plotted as the negative derivate of fluorescence F vs. T) analysis from a qPCR assay based on SYBR-green fluorescence for detection of PERVs. The dissociation temperature range extends from 65 to 90 °C. Melting peaks for the PERVs positive controls range 81.0 ± 0.5 °C. Melting peaks for the paraffin embedded lymph node samples range 80.5 ± 0.5 °C. No melting curve graphs are observed for negative control (human cell line) and NTC.

The transplanted kidney was surrounded by moderate edema and fibrin and its surface and cortex were diffusely pale. Ureters were moderately dilatated with thickened walls and, during their longitudinal dissection, a moderate amount of urine mixed to white fibrillar fibrinous material was seen. In the transplanted kidney in association with the histological features of a T-cell-mediated graft rejection (moderate to severe interstitial peritubular collection of small lymphocytes and mild to moderate tubulitis), also multifocal nodular aggregates with infiltrative growth of neoplastic cells were present, and were morphologically similar to the neoplastic population of lymph nodes often intermingled with small lymphocyte and plasma cells were apparent in both cortex and medulla (Figure 4(A)). The native kidney showed only a mild multifocal interstitial infiltration of lymphocytes and rare plasma cells (Figure 4(B)).

**Figure 4.**
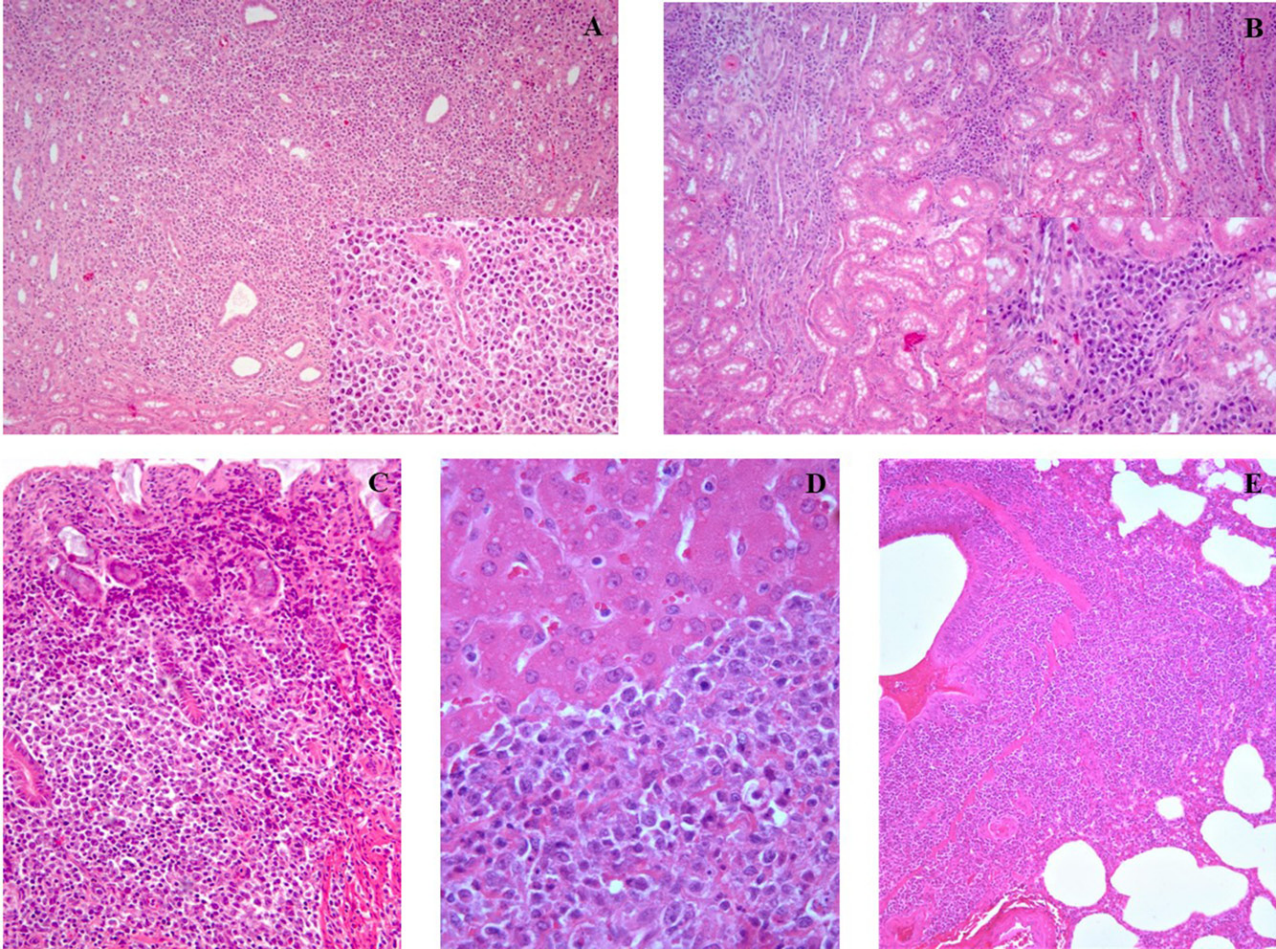
Transplanted kidney: nodular aggregates of neoplastic lymphocytes intermixed with small lymphocytes and plasma cells (A, H&E, 10x, inset 40x). Native kidney: mild interstitial nephritis characterized by infiltration of lymphocytes and rare plasma cells (B, H&E, 10x, inset 40x). Stomach: diffuse infiltration of the lamina propria by neoplastic lymphoid cells (C, H&E, 20x). Liver: lymphoid infiltration of the lobular septae (D, H&E, 40x). Lung: lymphoid infiltration of the bronchial associated lymphoid tissue (BALT) (E, H&E, 10x).

Other findings at necropsy included pulmonary congestion and edema, especially in cranial and ventral lobes, confirmed also by histopathology that let to appreciate also neoplastic lymphoid expansion of the BALT (Figure 4(E)). Gastric mucosa was thickened by diffuse nodular proliferation up to 2 cm in diameter with occasionally ulceration or navel-like shape (Figure 1(E)). Histologically the mucosa was diffusely ulcerated and the lamina propria densely infiltrated by neoplastic lymphoid cells (Figure 4(C)).

Grossly, in the liver there were multifocal areas of interstitial broadening that histologically were consistent with neoplastic lymphoid infiltration of the interlobular septae often associated with lobular invasion (Figure 4(D)). Mild pericardial and abdominal serous and hematic effusion were present. Hyperplastic chronic cystitis secondary to catheterization was also evident.

Immunohistochemistry on sections of lymph nodes, spleen, stomach and transplanted kidney showed a mixed population of CD79a positive (Figure 5(A)) and CD3 positive (Figure 5(B)) neoplastic lymphoid cells often intermingled with small well differentiated lymphocytes mainly CD3+. In the transplanted kidney, these latter were the main population in the interstitial reaction surrounding the tubules in areas lacking the neoplastic lymphoid infiltration.

**Figure 5.**
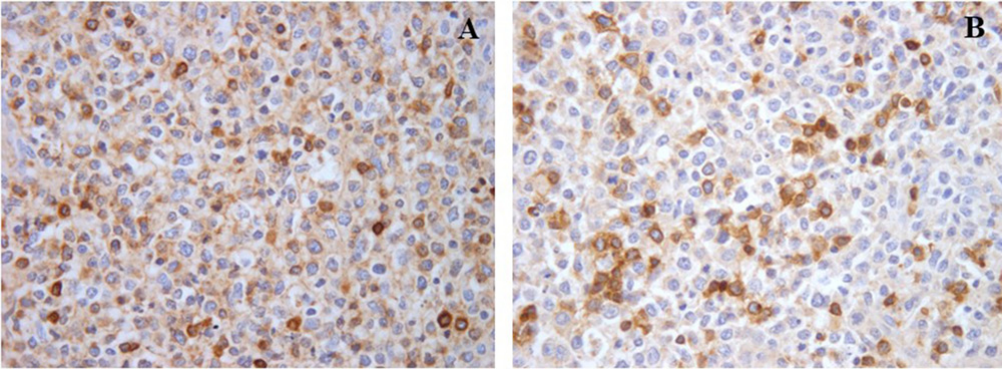
Lymph node: neoplastic cell population is composed by both CD79a (A, IHC for CD79, 40x) and CD3 (B, IHC for CD3, 40x) positive cells.

Based on the above findings, it had been concluded that one gilt out of 18 (6%) developed PTLD. PTLD is an abnormal lymphoid proliferation that occurs both in humans and animals (Schmidtko et al. 2002). Most cases of PTLD originate from B-lymphocyte disorders but rarely from T-lymphocytes and natural killer cells (Huang et al. 2001). In human medicine, PTLD is classified into four grades: early lesions, polymorphic PTLD, monomorphic PTLD and Hodgkin lymphoma (Swerdlow 2016; Campo et al. 2011; Jagadeesh et al. 2012). The disease often arises, both in humans and in animals, as a consequence of solid organs grafts: transplant recipients can be affected by PTLD caused by prolonged immunosuppression and concurrent viral infections (Mueller et al. 2004).

Human PTLD cases are strictly associated with Epstein–Barr virus (EBV) infection: 90% of post-transplant lymphomas occur in EBV + patients (Page et al. 2013). EBV tropism is oriented to B lymphocytes of the host. In immunocompetent hosts the infection of EBV is typically asymptomatic and evolves in two phases: the litic phase, characterized by sprightly proliferation of cytotoxic T lymphocytes, and the latent phase, characterized by less expression of B cells-antigen and virus immortalization in B cells (Green and Michaels 2013). The disease can be induced by latent virus reactivation in the recipient or by transmission via EBV infected donor cells.

In human transplantation, the majority of PTLD cases arise from the transmission of the donor EBV-infected B lymphocytes. PTLD can arise both in EBV positive and EBV negative recipients, but the risk is increased in EBV negative recipients. In EBV positive recipient immunosuppression leads to T-cells depression and possibly to an uncontrolled B-cells proliferation. In EBV negative recipient, the combination of immunosuppression and lack of EBV specific immunity, lead to an uncontrolled proliferation of EBV-transformed B lymphocytes (Paya et al. 1999; Al-Mansour et al. 2013).

In human polymorphic PTLD, most relevant histological features are mixed population of small and medium-sized lymphocytes, immunoblasts and plasma cells. Neoplastic infiltrate cause destruction and effacement of the involved tissues architecture. Atypia, necrosis and high mitotic rate are frequent (Tsao and Hsi 2007; Al-Mansour et al. 2013).

Although, clinical practice and many research studies demonstrate that EBV causes B-lymphocyte proliferation and non-Hodgkin’s lymphoma, the exact role of EBV in this process is still not clear (Doucette et al. 2007).

As aforementioned, PTLD can be divided into four groups based on morphologic, immunophenotyping and molecular characteristics (Swerdlow et al., 2016). The early lesions type of PTLD is the only type of PTLD in which the lymph node architecture is maintained. It is characterized by the invasion of plasma cells and both B and T immunoblasts in the interfollicular region. In polymorphic PTLD both the lymph nodes and extra-nodal tissue architecture is destroyed and replaced by lymphocytes, plasma cells and both B and T immunoblasts. Within the neoplasm necrosis and a high mitotic count can be observed. Monomorphic PTLD is characterized by a uniform population of B or T lymphocytes, hence, it can be subclassified as B-cell or T-cell neoplasm. The last type of PTLD is a classical Hodgkin lymphoma, which has mixed B and T cellularity (Jagadeesh et al. 2012). In the present case, based on cellular morphology, mixed B and T phenotype of the neoplastic lymphoid cell population, and involvement of both lymphoid (lymph nodes, spleen, BALT) and non-lymphoid organs (stomach, liver), the diagnosis was polymorphic PTLD, according to the WHO classification (Swerdlow et al. 2016).

Differences are known between human and porcine histological architecture of lymph nodes; B-cells germinal centers are located in the interior of the lymph node, and cortical and paracortical region are thicker than in humans (Zimmerman et al. 2012). Notwithstanding these differences in lymphoid system human and swine present anatomical, physiological and tumorgenesis similarities (Adam et al. 2007) in lymphoma and swine can be considered a model also for the development of human PTLD.

Porcine PTLD is a recently discovered disease, developing in swine undergoing allogenic transplantation (Huang et al. 2001). The majority of porcine PTLD cases are associated with PLHV-1 infection but a small proportion of cases develop with no evidence of infection (Dor et al. 2004; Doucette et al. 2007). Other viruses, such as Porcine endogenous retroviruses (PERVs), have been associated to lymphoma after transplantation in swine (Fishman 2018).

To the best of the author’s knowledge, this is the first PTLD case reported in a non-miniature breed of swine. In the current case, PTLD occurred 30 days after renal transplantation, similarly to previous reports in which PTLD was detected from 21 to 45 days after the graft (Huang et al. 2001; Matar et al. 2015). However, the present case differs from previously reported cases with regards to the phenotype and tumor distribution. A monomorphic type of mainly B-cells with a localization restricted to the lymhoid tissue is predominantly described in previously reported PTLD (Huang et al. 2001; Cho et al. 2004; Dor et al. 2004), while in the present case the tumor has a mixed B and T cellular type and has spread to other non-lymphoid organs (lung, liver and stomach). The difference might be related to breed predisposition (miniature versus large pigs) to develop the two different forms of the disease. Lymphoid proliferation of the case here presented shares similar histological features with those of polymorphic human PTLD.

Lymphoid neoplasm in swine are rare entities and lymphoma is reported to be the most frequent. Lymphomas are classified as lymphocytic, lymphoblastic, histiocytic and mixed type (Ogihara et al. 2012). In this specific case, lymphoid proliferation share similarities with mixed type lymphomas: mixed small and large lymphocytes intermixed with plasma cells and effacement of extra-nodal tissues architecture. Development of these neoplastic lesions after immunosuppression and consequent solid organ transplantation, leads us to classify our case as PTLD.

The etiology of porcine PTLD is still unclear but, according to literature, both PHLV-1 and PERVs seem to be involved (Denner 2016). Previous studies demonstrate high incidence of PTLD in miniature swine undergoing xenotransplantation and a close relation between the disease and PLHV-1 infection (Huang et al. 2001; Cho et al. 2004).

PLHV-1 is a γ-herpesvirus infecting more than 80% of commercial pigs (Ehlers et al. 1999). Its genome is homologous to those of human EBV and human herpesvirus 8 (HHV-8), which are closely related to post-transplanted diseases in humans (Santoni et al. 2006). E4/BALF1h gene of PLHV-1 encodes a viral bcl-2 homologue from the same genomic position of EBV (BALF1). Both viral bcl-2 and the protein coded by BALF1 gene are reported to avoid apoptosis in viral infections (Goltz et al. 2002). Another important feature of PLHV-1 infection is the delay between virus replication and clinical disease: pharmacological greater intensity of immunosuppression, in association with the presence of PLHV-1, appears to activate latent viral infection and impairs T-cells function, leading to abnormal B-cells proliferation (Dor et al. 2004; Brema et al. 2008).

Porcine endogenous retroviruses (PERVs) have also been associated to lymphoma after transplantation (Fishman 2018) they are integrated in the pig’s genome and in immunosuppressed patients, their activation may induce tumor and/or immunodeficiency (Denner 2016, 2017). In literature, three subtypes of PERVs have been described: PERV-A and PERV-B, which can infect cells of different species (including humans) and PERV-C, which can infect pig cells only (Sypniewski et al. 2005; Bittmann et al. 2012).

Both PERVs and PLHV-1 can induce immunodeficiency and tumors, inhibiting T cells and overstimulating B cells (Page et al. 2013; Denner 2016). Awaiting to have further confirmation on the role of the two viruses in the etiopathogenesis of PTLD in swine, it appears that PTLD has a swine model of disease. Possibly, the case presented here (PERV but not PLHV-1 associated) parallels those cases of human PTLD in which EBV is not the cause. The risk of developing the disease should be considered during experiments of transplantation in the porcine species. Furthermore, its management can be a useful model for the human counterpart of the disease.

Both PHLV-1 and PERVs are common infections in conventional pig herds and their exact role on tumour’s pathogenesis in the porcine species still has to be elucidated. In this study, we described the histopathology, immunohistochemistry, and molecular biology of a case of PTLD in a swine occurring during an experiment of renal transplantation.

## Supplementary Material

Supplemental data for this article can be accessed at https://doi.org/10.1080/01652176.2019.1661542.
