# Supplementary material for: Polymorphic post-transplant lymphoproliferative disorder in a gilt
Source: Vet Q. 2019 Oct 1;39(1):136–42. doi: 10.1080/01652176.2019.1661542 (PMC8923020; doi:10.1080/01652176.2019.1661542)
Supplement: Supplemental data for this article can be accessed at https://doi.org/10.1080/01652176.2019.1661542. [file TVEQ_A_1661542_SM8225.docx]

PTLD Post Transplanted lymphoproliferative disorder

qPCR Real time polymerase chain reaction

PERVs Porcine Endogenous Retroviruses

PLHV-1 Porcine Lymphotropic Herpesvirus 1

BALT Bronchial Associated Lymphoid Tissue

EBV Epstein-Barr Virus
